# Anterior six arms prolene mesh for high stage vaginal prolapse: five years follow-up

**DOI:** 10.1590/S1677-5538.IBJU.2016.0482

**Published:** 2017

**Authors:** Luis Gustavo M. de Toledo, André Costa-Matos, Susane Mey Hwang, Raquel Dória Ramos Richetti, Silvia S. Carramão, Antônio P. F. Auge

**Affiliations:** 1 Faculdade de Ciências Médicas da Santa Casa de São Paulo, São Paulo, SP, Brasil;; 2 Serviço de Uroginecologia, Maternidade Cachoeirinha, São Paulo, SP, Brasil

**Keywords:** Surgical Mesh, Pelvic Organ Prolapse, Surgical Procedures, Operative

## Abstract

**Introduction:**

In high stage vaginal prolapse, recurrence risk patients, anterior and apical defects need to be addressed in the same procedure. The pre-molded commercial mesh kits are expensive and not always available. Alternative effective and safe treatment ways, with lower costs are desirable.

**Objective:**

To present long term follow-up of patients treated with a homemade mesh shape to correct high stage prolapses.

**Materials and Methods:**

We describe prospectively 18 patients with anterior and apical vaginal prolapses, stages III and IV, repaired using this specific design of mesh. All patients were submitted to pre-operative clinical evaluation and urodynamics. Prolapse was classified using the pelvic organ prolapse quantification (POP-Q). Intervention Prolapse surgery, using a six arms prolene mesh, through a single anterior vaginal incision. Outcome Measurements: POP-Q, patients satisfaction, descriptive statistical analysis.

**Results:**

Between February 2009 and Oct 2010, 18 consecutive women underwent the above-mentioned surgery. Mean age was 68 years. At a mean follow-up of .,4 years (5 to 5.8 years), 16 (89%) patients were continent, mean Ba point came from +4.7cm to - 2.5cm, mean C point from +2.8cm to -6.6cm and mean Bp point from +1.3 to -1.7cm. There were two (11%) objective failures, but all the patients were considered success subjectively. There were two cases of mesh vaginal extrusion.

**Conclusions:**

The homemade six arms prolene mesh allows concomitant correction of anterior and apical prolapses, through a single anterior vaginal incision, being an effective, safe and affordable treatment option when mesh is needed.

## INTRODUCTION

Urinary incontinence and pelvic organ prolapse are some of the most commonly treated conditions in postmenopausal women. American women have an estimated 20% lifetime risk of undergoing a surgery for urinary incontinence or pelvic organ prolapse (POP) (1). Surgical cure rates vary greatly depending on surgical technique and the type of materials used (2).

According to Whiteside et al. (3), 58% of the women who had undergone surgery for genital prolapse, presented recurrence at 1-year follow-up evaluation. Treatment failures could be attributable to the use of weak native tissues. The use of mesh in POP surgery has been discussed extensively lately, but the benefit is likely when there is a combination of risk factors such as recurrent POP, deficient fascia, chronic increased abdominal pressure, advanced stage and apical-anterior defect (4).Surgery using mesh, in this situation, presents better results in correcting high grades anterior and apical vaginal prolapses (2, 5).

There is a high concomitance of apical and anterior prolapses in POP-Qstages III and IV. High stage anterior prolapses, those that may have benefit from mesh, are almost never just an anterior defect. Apical prolapse is frequently present and may be unnoticed. When apical prolapse is predominant, it’s correction alone leads to anterior recurrence in up to 40% cases (6). Therefore, correction of these two defects, simultaneously, is required for a successful treatment. Today, abdominal sacral colpopexy using synthetic mesh over the anterior and/or posterior vaginal wall seems to be the more reliable procedure for the cure of genital prolapse with vaginal vault involvement, but the latest publications showed similar results with sacrospinous fixation that also provides good vaginal support (5, 7-9).

Previous experience has showed that synthetic material can reduce prolapse recurrence rates (10). The last generation of mesh kits were introduced addressing this practical problem, the combination of anterior and apical prolapses. Other ways of surgical management of multi-compartment prolapses are described. Feiner et al. (11) presented good results using anterior mesh associated to sacrospinous ligament sutures to treat concomitant anterior and apical defects. Mourtialon and Delorme (12) have proposed a similar mesh shape with good initial results. We propose a mesh shape to correct both anterior and apical prolapses, associated or not with stress urinary incontinence (SUI), with a single anterior vaginal incision, showing long-term results.

## PATIENTS AND METHODS

**Figure 1:**
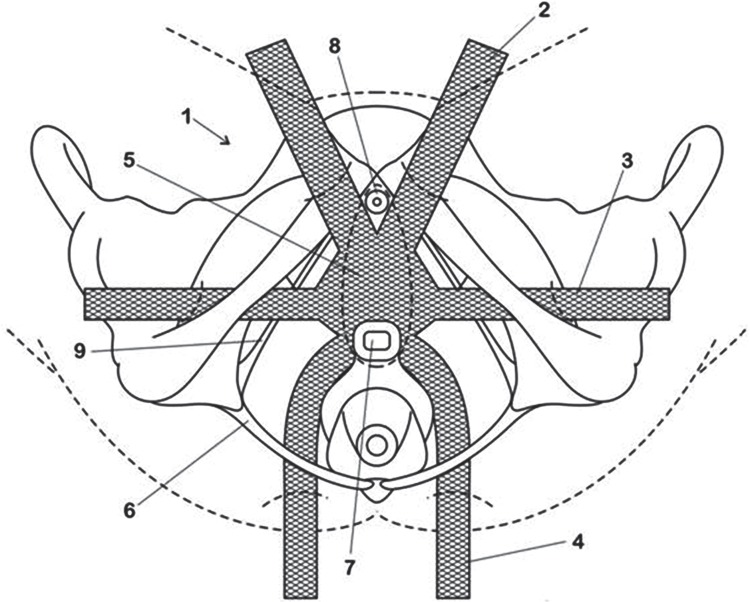
Antero apical mesh configuration: 1) the mesh;2) pre pubic arms;3) transobturator arms;4)sacrospinous ligament arms;5) body of the mesh;6) sacrospinous ligament;7) uterus cervix or vaginal vault;8) urethra;9) arcus tendineus.

Patients withanterior and/or apical stage III or IV POP and not needing hysterectomy for any uterine pathology met the inclusion criteria. All patients who met inclusion criteria and were treated surgically from February 2009 to Oct 2010, by two of the authors, in an institutional referral center of São Paulo, Brazil, were included. We describe prospectively 18 consecutive patients who were surgically treated using a specific design of mesh (Figure-1). All patients were operated by the resident physician in training and were supervised and assisted by two surgeons (LGMT and ACM). The surgical steps were standardized so as to minimize variations between the two surgeons. The Institutional Review Board approved the study protocol, and a written informed consent was obtained after giving detailed explanations about the procedure. No financial assistance was received from any company or institution for the execution of this study.

All patients were submitted to a complete pre-operative evaluation including medical history, physical examination and urine culture. Urodynamic evaluation was performed when indicated by urinary symptoms (urinary incontinence, urgency or voiding disorders) or positive cough stress test (spontaneous or after prolapse reduction). Urodynamic investigation included a free flowmetry, filling cystomanometry and pressure flow studies(13). Occult SUI was assessed by prolapse reduction test with Cheron clamp, in supine and standing positions. The prolapse stage was assessed in lithotomy position while patient performed Valsalva maneuver. Prolapse was classified using Pelvic Organ Prolapse Quantification (POP-Q) (14). International Consultation on Incontinence Questionnaire-Vaginal Symptoms (ICIQ-VS) was used to subjective assessment of POP bother, severity, and impact on quality of life (15). Mesh complications were classified by IUGA/ICS Prosthesis/Graft Complication Classification Code (16).

A concomitant procedure was performed if necessary, including posterior vaginal repair and perineorraphy. Slings were not used as the anterior arms of the mesh were used to give a mid urethral support.

After surgery, evaluations were done at 3 weeks, 3 and 6 months and annually thereafter. Objective recurrent prolapse was defined as any anterior or posterior descent of stage II or higher (Ba or Bp≥-1cm), or apical descent of more than 1/3TVL (total vaginal length), even if asymptomatic. The post-operative assessments were performed by a third staff member (SMH).

### Surgical description

This procedure was performed under spinal anesthesia. All patients were placed in the lithotomy position with thighs flexed at approximately 90º. After cleaning the entire surgical area with antiseptic, a Foley 16Fr catheter was placed. All patients had an intravenous perioperative antibiotic prophylaxis (Cefazolin 2g). We infiltrate the vaginal wall with a vasoconstrictive (epinephrine 1:200.000) solution to ease dissection and reduce bleeding. A midline longitudinal incision was made in the anterior vaginal wall from 2cm below the urethral meatus until 2cm from the uterine cervix. Sub fascial mucosal dissection was done until arcus tendineus bilaterally, pericervical ring proximally and middle urethra distally. Midline plication was done to reduce cystocele. Mesh positioning was done with aid of permanent needles, delivering two pre-pubic arms providing sub urethral support and avoiding mesh migration proximally, two transobturator (TO) arms as close as possible to the ischial spine through the arcus tendineus ligament, treating lateral defect, and two arms through the sacrospinous ligament, through trans gluteus access, 1.5 to 3cm medial to the ischial spine, to avoid injury to the pudendal nerves and vessels, rounding anteriorly the uterine cervix, to treat apical support defect. Fixation of the proximal end of the mesh to the pericervical ring used 2.0-polyglactin absorbable sutures (Vicryl, Ethicon, Somerville, NJ, USA). The vaginal incision was closed with interrupted 3.0-polycaprone sutures, before pulling the gluteal arms and prolapse reduction. One of the gluteus arms was pierced to the other side through retro anal subcutaneous path, and fixed to the other arm, with absorbable suture, to prevent loosening and early relapse. After the procedure, lubricated vaginal packing was placed for 12hs (Figure-1).

**Figure 2:**
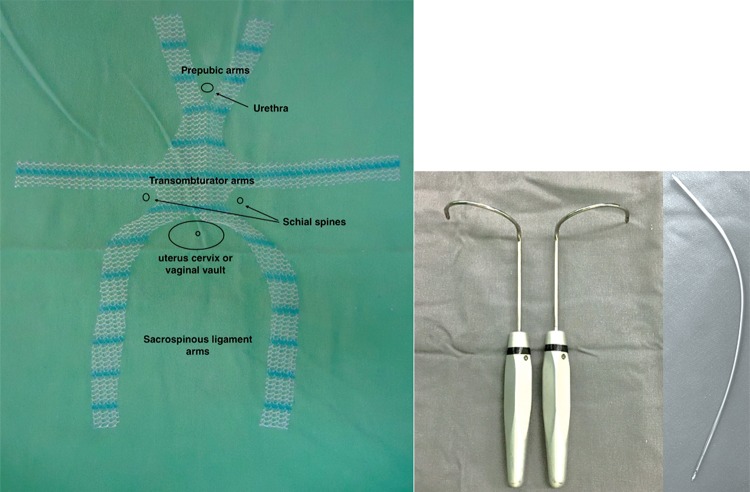
Molded anterior apical mesh, transobturator and trans gluteus sacrospinous retrieving needles.

The mesh used was a non-absorbable monofilament soft polypropylene mesh (Gynecare Gynemesh™ , Ethicon, Somerville, NJ, USA). Permanent retrieving needles were used to place the mesh arms (Figure-2).

Simple descriptive statistical analysis is described.

## RESULTS

Nineteen patients were operated using the six arms mesh during the study period, one of then lost follow-up and could not be reached for five years outcomes. Eighteen women had their data analysed. Mean age was 68 years. Pre-operative patient’s characteristics are summarized in Table-1. Six (33%) patients had previous abdominal hysterectomy, and two had previous POP surgery. Three patients (16%) complained of SUI before surgery, seven had urgency (39%) and eight patients (44%) presented some voiding dysfunction (inability to empty the bladder, poor stream, digital maneuvers). Pre-operative clinical data are listed in Table-2.

**Table 1:** Pre-operative patient’s characteristics.

Characteristics		Mean (range) or n (%)
Age (years)	68 (53-81)	
Parity (n)	5 (1-13)	
BMI	27 (16.8-33)	
Menopausal status	18 (100%)	
Hormone replacement therapy	None	
Previous hysterectomy	6 (33%)	
Previous prolapse repair	2 (11%)	
Previous surgery for SUI	2 (11%)	
Sexual activity	2 (11%)	
Dyspareunia	2 (11%)	

**BMI =** Body Mass Index

**Table 2:** Pre-operative POP-Q stage data.

Prolapse	Anterior Vaginal Wall	Apical	Posterior Vaginal Wall
Stage 0–1	None	None	6(33.3%)
Stage 2	None	4 (22.2%)	4 (22.2%)
Stage 3	10 (55.6%)	10 (55.6%)	7(38.9%)
Stage 4	8 (44.4%)	4 (22.2%)	1 (5.6%)

**POP-Q =** pelvic organ prolapse quantification

Average operative time was 132min (90-180min). Posterior colporrhaphy was performed in six (33%) patients. No bladder or rectal injury was recorded. One patient had self-limited hypotension during legs repositioning. One patient had hemorrhage requiring transfusion of 3UI of blood concentrate, and presented with pelvic hematoma requiring open surgical drainage by Pfannenstiel incision. Three patients had urinary retention needing to be discharged with urinary catheter; two of them needed surgical relaxation of the pre pubic arms a week later, which could be done with local anesthesia. They regained normal voiding. The others had catheter removed after 24h. Median catheter use was one day (1-7d). Ten (55%) patients went home in the first postoperative day. Median hospital stay was oneday (1-18d). Mesh vaginal extrusion was identified in two patients, three months after surgery. They were two and three cm in diameter and occurred in the suture line. The patients presented with vaginal discharge. Both were classified as 3BbT3S1 and were successfully treated with extirpation of the extruded mesh, with no prolapse recurrence. No organ erosion was identified.

At a mean follow-up of 5.4 years (5 to 5.8yr), 16 (89%) patients were continent, mean Ba point moved from +4.7cm preoperatively to -2.5cm postoperatively, mean C point, from +2.8cm to -6.6cm and mean Bp point, from +1.3 to -1.7cm (Table-3). All the patients were considered success subjectively. There were two objective relapses, one apical stage I and one posterior stage II, both asymptomatic, with no need of reoperation so far. The two sexual actives patients had no dyspareunia. SUI was diagnosed in three patients preoperatively and was totally controlled after surgery in two of them. Five (28%) patients complained of urgency and three (16%) of voiding symptoms postoperatively. The mean ICIQ-VS scores improved significantly postoperatively in all three domains. Vaginal symptom scored from 36.3 to 7.8; sexual matter scored from 30 to 12 (only two patients) and quality of life scored from 9 to 1.6.

**Table 3:** Pre and postoperative POP-Q.

Mean pre and post operative POP-Q				

Point	Mean pre-op (cm)	Mean post-op (cm)	Mean difference (cm)	P value
Aa	1.5	-2.5	4.0	<0.001
Ba	4.7	-2.5	7.2	<0.001
Ap	0	-1.7	1.7	NS
Bp	1.3	-1.7	3.0	0.02
C	2.8	-6.6	9.4	<0.001
D	1.4	-7.8	9.2	<0.001

**POP-Q =** Pelvic organ prolapse quantification

## DISCUSSION

With an aging population, the demand for physicians and surgeons trained in management of pelvic organ prolapse will increase. New technologies such as the development of vaginal approaches, using or not prosthetic devices, which are effective and reproducible may facilitate surgery and provide more widely better results.

The sacrocolpopexy is more widely applied for level one and high grades prolapses, with long follow-up consistent results and thus this technique is considered the gold standard(9, 17, 18). However, data of transvaginal sacrospinous colpopexy using mesh, with up to 90% objective cure and low complications rate, with relative long follow-up have also been published (19, 20). A recently published systematic review, comparing transvaginal meshes with native tissue repair for vaginal prolapse reported that permanent meshes are associated with lower rates of subjective and objective prolapse recurrence, and prolapse reoperations. But, on the other hand, it is also associated to 8% reoperation for mesh exposure, more bladder injury and de novo stress urinary incontinence (21). We understand and reinforce that patient selection is extremely important to avoid unnecessary mesh use. We also believe that most mesh complications are due to technical mistakes, such as excess of mesh volume, focal tension, asymmetry, folding, mucosal damage and ischemia during dissection. Another practical observation is that we can lower complications maintaining the results using less and less mesh volume, prioritizing the correction of apical defect, as an anterior apical sling, pushing uterine cervix or vaginal vault towards sacrospinous ligament. We feel this is the right way of high stage POP treatment, less mesh volume addressing apical defect.

The anterior vaginal approach to the sacrospinous ligament is not a new technique, but its association to mesh molded to treat concomitantly apical and anterior vaginal prolapses, has recently been developed. By the time our patients were treated, only Elevate^TM^ (American Medical Systems, Minnetonka, Minnesota, USA) had been marketed. The older generation of meshes kits used to treat apical defect are combined with posterior prolapse treatment, which is unnecessary since the later has no benefit from meshes, presenting similar results with conventional approach (9, 11). On the other hand, stages III and IV anterior prolapses are rarely isolated and generally associated with apical defect. So, it’s rational that mesh treatment should combine anterior and apical repair and not posterior and apical as most of the mesh kits used to be designed for.

In the end of 2009, Mourtialon and Delorme (12) have published a new approach to fix cystocele and uterus or vault prolapsed with a six arms mesh that allow, as we do, only one anterior incision. However, a small difference is that we use pre-pubic arms, which prevent proximal migration, stabilizes the mesh distally preventing its folding in the longitudinal direction, and can eventually treat SUI. Importantly, thesepre-pubic arms differfrompre-pubic slings, which were abandoneddue to complicationssuch as extrusion and failures by migrating distally. In the pre-pubic sling, the force vector is toward the urethral meatus because there is no traction in the proximal direction. In other way, the anterior apical mesh is fixed proximally to the pericervical ring and to sacrospinous ligaments which prevents distal migration.

**Figure 3:**
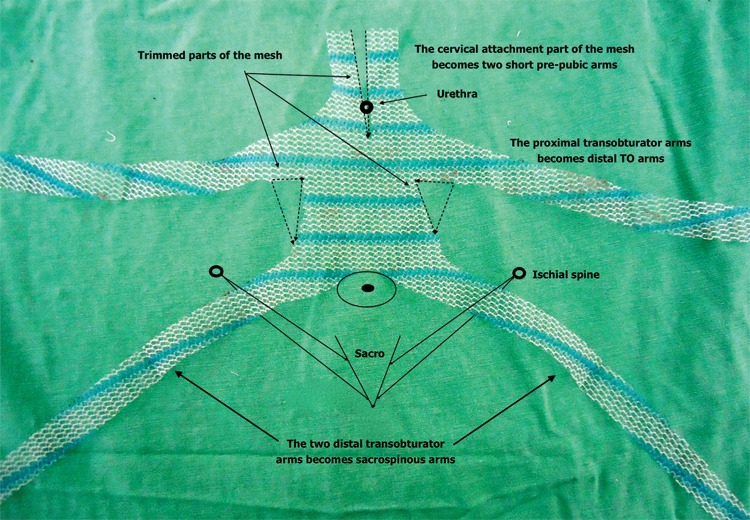
Inverted Anterior Prolift.

Alternative ways of treating multi-compartment prolapse are described. Feiner et al. (11) presented good results using anterior mesh associated to sacrospinous ligament sutures to treat concomitant anterior and apical defects. At the time, we also had done adaptations to treat these kinds of prolapse, one of them was, as we called it, the “Inverted Anterior Prolift”. We used it upside down, so the two distal TO arms transfixed sacrospinous ligament through trans gluteal approach, the proximal TO arms becomes distal and cervical attachment part of mesh becomes two short pre-pubic arms (Figure-3).

Furthermore, the choice between a complete vaginal reconstruction of all compartments and a specific repair of only the defective areas is much debated (8). The risk of a specific repair is to provide a de novo prolapse in a compartment that previously appeared well supported. According to the prospective epidemiologic evaluation of Clark et al. (6), 60% of recurrence occurred at the same anatomic site, implying direct failure of the surgical procedure and 40% of recurrence occurred at a different site, which suggests a change in stability of the pelvic floor after surgery.

Our design of mesh allows correction of the three De Lancey´s level defects using only one incision and low volume of mesh. Therefore, anterior apical mesh seems rational and should reduce surgery time, morbidity, relapses and costs in high grade, recurrence risk prolapse patients.

Despite not removing vaginal epithelium, at the short-term follow-up, the vagina adheres to the underlying mesh, providing good functional and anatomical results while avoiding vaginal narrowing or shortening in most cases. Milani et al. (22) reported increasing dyspareunia with the anterior and posterior prolapse repair with prolene mesh. Dyspareunia increased by 20% after anterior repair and by 63% after posterior repair. Our findings don’t suggest such results, however the majority of our patients (89%) were sexual inactive. Dwyer and O’Reilly (23) published sexual outcomes after transvaginal repair with Atrium polypropylene mesh (Hudson, New Hampshire, USA) in 67 sexually active patients. Dyspareunia was reported by 25.8% of the patients before surgery and 9.1% of the patients at 24 months post-operatively. Only three cases of de novo dyspareunia were reported in this study. The authors believe, as we do, that the removal of excess vaginal tissue is unnecessary and indeed deleterious.

Limitations of our study are the small sample size, poor LUTS assessment and the absence of a control group. However, five years follow up gives consistency to our good results and low complication rates. Some issues need to be addressed in future studies including prospective randomized comparison of anatomical and functional outcomes for mesh reinforcement versus site specific fascial repair alone and abdominal sacrocolpopexy. Another issue to be answered is if minimal (sling) apical mesh lowers complications maintaining results better than native tissue repair.

## CONCLUSIONS

The six arms prolene mesh allows concomitant correction of anterior and apical high stages prolapses, through a single anterior vaginal incision, with high success and acceptable complications rates. It may be an alternative when mesh is desirable but kits are not available.
